# Understanding rat emotional responses to CO_2_

**DOI:** 10.1038/s41398-020-00936-w

**Published:** 2020-07-24

**Authors:** Lucía Améndola, Daniel. M. Weary

**Affiliations:** Animal Welfare Program, University of British Columbia, 2357 Main Mall, Vancouver, BC V6T 1Z4 Canada

**Keywords:** Neuroscience, Psychology

## Abstract

The aim of this review is to summarize evidence regarding rat emotional experiences during carbon dioxide (CO_2_) exposure. The studies reviewed show that CO_2_ exposure is aversive to rats, and that rats respond to CO_2_ exposure with active and passive defense behaviors. Plasma corticosterone and bradycardia increased in rats exposed to CO_2_. As with anxiogenic drugs, responses to CO_2_ are counteracted by the administration of anxiolytics, SRIs, and SSRI’s. Human studies reviewed indicate that, when inhaling CO_2_, humans experience feelings of anxiety fear and panic, and that administration of benzodiazepines, serotonin precursors, and SSRIs ameliorate these feelings. In vivo and in vitro rat studies reviewed show that brain regions, ion channels, and neurotransmitters involved in negative emotional responses are activated by hypercapnia and acidosis associated with CO_2_ exposure. On the basis of the behavioral, physiological, and neurobiological evidence reviewed, we conclude that CO_2_ elicits negative emotions in rats.

## Introduction

Several behavioral studies indicate that carbon dioxide (CO_2_) elicits negative responses and is aversive to rats^1–8^ although not all studies agree^9,10^. Rat exposure to CO_2_ is a well-accepted translational model for the understanding of fear, anxiety, dyspnea (feeling of breathlessness), and panic in humans^11^.

Pain, fear, panic, and anxiety are considered high arousal, negatively valenced emotional states. Here, we follow a functional working definition that identifies emotional responses as objectively observable, and feelings of emotions as the conscious awareness of emotions experienced as positive or negative^12,13^. Emotions are “central states” inferred from brain arousal, and behavioral and physiological changes due to the presentation of a competent situation-dependent stimuli^12^. Not all stimuli elicit an emotional response; the competence of the stimuli will depend on the individual’s evolutionary history (innate response), personal experience (developmental plasticity and learning), discriminative properties of the stimuli (intensity and type of stimuli), and the current situation (e.g., controllability). Induction regions in the brain are responsible for the emotional cascade (chemical and neural reaction) that lead to the execution of appropriate behavioral, physiological, and brain responses to cope with a competent stimuli^12^.

Feelings of emotions can be described using a two-axis (arousal/valence) model^14^, and comprise different patterns of neural, behavioral, and physiological responses^15^. In the scientific literature, there is little consensus regarding what constitutes the feelings of emotions, and how and where these feelings are evoked in the brain. For example, some argue that this requires an internal self-representation of body and mind changes (i.e., interoception; homeostatic state, state of preparedness to cope, and motivational state) that accompany emotions, a process thought to occur in cortical areas of the brain (e.g., insula and cingulate cortex). Within this view, species that possess interoception could feel emotions^12,15–17^. Other authors emphasize the role of neocortical working memory (i.e., temporary hold and manipulation of information while doing mental work) as a requirement for feeling emotions, an idea that may exclude some nonhuman animals^18^. Panksepp^13^ argued that basic neurobiological subcortical areas present within all mammals are responsible for both emotion and feelings.

The assessment of felt emotions remains a major challenge^13,19^, but strong inferences about felt experiences can be made through a combination of evidence from (1) central state emotions (i.e., regional and local brain arousal, and behavioral and physiological changes when a competent situation-dependent stimuli is present)^12,15,20^, (2) indications of awareness (i.e., behavioral plasticity, direction and maintenance of attention, and agency)^19^, (3) functional homology in which human felt experiences and their associated physiological, neural, and behavioral responses can be cautiously compared to that of animal responses to the same stimuli and used as a proxy to the animal’s felt emotions^13,21^, and (4) drug treatments that target specific feelings of emotions in humans to infer specific feelings in animals^19^.

The aim of this paper is to review the available information on the behavioral, physiological, and neurological effects of CO_2_ on rat emotional responses, discussing inconsistencies between studies. We also cite research on human felt experiences when inhaling CO_2_ as context for understanding of rat emotional experiences. We focus on rats given the mounting research done in this species, but also refer to mouse studies when appropriate. This review includes only articles that were peer-reviewed, written in English, that assessed the effects of CO_2_ inhalation (or hypercapnia), and that report at least one outcome (behavioral, physiological, or neurobiological) indicative of an emotional response. Throughout the review, we describe the magnitude of effects only when studies reported these as statistically significant.

## Biological responses

Inhaling high concentrations of CO_2_ suppresses the removal of metabolic waste CO_2_. The increase in CO_2_ metabolic waste results in high arterial PaCO_2_ (hypercapnia), decreasing blood pH (acidosis; pH < 7.2)^22^. Since acid–base and PaCO_2_ balance are important for survival, mammals possess mechanisms of detecting changes in pH and CO_2_, and responding to these threats to homeostasis. A rise in arterial PaCO_2_ and decrease in pH are detected by peripheral and central chemoreceptor cells. A synergic output from the integration of peripheral and central chemoreceptor inputs^23^, adjusts the ventilatory response according to the blood gas stimuli^22^. For example, when exposed to the 20% CO_2_ challenge (rapidly increasing concentration stabilizing at 20% CO_2_ after 5 min), rats showed increased breathing frequency from 100 to 130 breaths per minute (b.p.m.) within the first minute, and 130–165 b.p.m. after 2 min of exposure^24^. Similar physiological responses are found in humans; when subjected to inhalation of 5–7% CO_2_, healthy humans increase both tidal volume and respiratory frequency as a compensatory mechanism to remove excess CO_2_^25^.

By modulating ventilation, mammals can cope with slight increases in CO_2_, but when increased ventilation is not sufficient to remove excess CO_2_ the animal may be experiencing negative emotional states, such as air hunger^21^, as evidenced by behavioral and physiological responses, and brain activation.

### Behavioral responses

Here, we discuss the behavioral evidence of negative emotional states in three sections: (1) studies where animals cannot escape exposure to CO_2_, (2) studies where animals can avoid exposure to CO_2_, and (3) other situations where behavior is used to infer negative emotional states during or after exposure to CO_2_.

#### Inescapable exposure to CO_2_

To assess emotions evoked by CO_2_, one common approach is forced exposure to the gas^6,9,26^. The working (and typically implicit) assumption is that the frequency, duration, and intensity of the response reflects the intensity of the rat’s negative emotional experience to the procedure.

Several studies have found behavioral evidence of negative states in rats exposed to CO_2_. Niel and colleagues^6,7^, using ~17% CO_2_ chamber vol. min^−1^, found that rats showed increased frequencies and intensities of several behaviors associated with distress. The onset of rearing and increased locomotion occurred at ~5% CO_2_ and peaked at ~20% CO_2_. Escape behaviors (i.e., pushing and scratching at the lid) were observed at between 20 and 28% CO_2_. These results are consistent with others using slightly higher flow rates (18.5 and 23% CO_2_ chamber vol. min^−1^)^27,28^. Vocalizations in the range of 6–103 kHz have been reported for rats exposed to CO_2_ flow rates between 17 and 30% chamber vol. min^−1^ (refs. ^2,6^). However, one other study reported that rats exposed to 10% CO_2_ chamber vol. min^−1^ did not vocalize, and that locomotion and rearing did not increase relative to baseline levels^29^.

Rats exposed to the 20% CO_2_ challenge show variable results. Some studies have reported increases in locomotion but not freezing^24^, and others have found the reverse^30^. These results suggest that the type of defense behavior varies (between active and passive), but that some response is usually present.

When exposed to high concentrations of static CO_2_ (CO_2_ > 97%), some studies have reported that rats are less active and do not show struggling, vocalizations^9^, or other signs of distress^31^, but others have reported signs of asphyxia and behavioral excitation^3^. In these studies, however, behaviors were sometimes recorded without baseline or acclimation periods^9^, and behavioral responses were not clearly defined. For example, “head rising” was described as “inquisitive or agitated movements of head,” vocalizations as “squealing and other noises,” and escape as “attempts to get out of the box”^31^, or responses were simply mentioned without description^3^. Without control animals, baseline observations, and a clear description of behaviors, interpretation of these results is challenging.

Strain differences in rat responses to CO_2_ have been seldom assessed. Winter and colleagues^32^ showed that exposure to 10% static CO_2_ elicits freezing behavior in Long Evans and Wistar Kyoto strains, but not in Sprague Dawley and Wistar strains. Sprague Dawley rats often respond to CO_2_ exposure by increased active defense behavioral responses^2,6,24,27,28,33^, but the absence of responses has also been also reported for this strain^29,31^. In contrast, Lister Hooded rats decrease activity during CO_2_ exposure^26^. Blackshaw et al^9^. reported a decrease in activity by Wistar rats during exposure to CO_2_, a result that differs from the findings of Niel et al^7^. Fisher rats—a strain selected for lower activity—showed no behavioral signs of distress when exposed to CO_2_ gradual fill^10^. In comparison to Sprague Dawley, Brown Norway rats show more digging in response to CO_2_ exposure^33^. These results suggest that strain differences limit comparability among studies.

Most CO_2_ exposure studies have used male rats. Male rats responded with increased active defense in some studies using forced exposure to CO_2_^3,6,7,24,28,34^, but other studies report no changes in behavior^10,29,31^. Female rats vocalized^2^ and increased active defense behaviors^27^ during CO_2_ forced exposure, but in another study showed no change in behavior^9^. Two studies recently published found no differences in behavioral responses to CO_2_ between male and female rats^33,35^.

Individual differences in rat responses to forced exposure to CO_2_ are often mentioned. It has been reported that only 20% of rats climbed the cage and 20% circled (i.e., moving around the perimeter of the cage)^31^, some rats expressed little and others numerous escape behaviors^6^, and only half of the rats tested increased locomotion^7^. Variation in rat response to CO_2_ may be related to reactivity^36^. For example, it has been shown that individual differences in active defense behaviors are consistent between two forced exposures to CO_2_ (ref. ^27^).

In summary, there is considerable variation in responses of rats to forced CO_2_ exposure within and between studies. Strain, sex, and individual differences account for some of this variability, but contrasting results within the same sex and strain still exist. Within-study variation in rat responses could be reflective of individual differences in CO_2_ reactivity. Differences in methodology, including the use of baselines, controls, type of cages, and induction method (e.g., static versus gradual fill, variable concentrations, and flows rates), and the lack of well-defined behaviors and interpretation of behaviors limit comparability between studies. Nonetheless, considering the responses summarized in Table 1, it seems likely that rats exposed to CO_2_ experience negative emotional states when escape is prevented.

**Table 1:** Forced exposure studies specifying delivery methods, concentration or flow rate used, strain and sex, whether the study contained baseline and well-defined behavioral categories, and a summary of results.

Strain	Delivery method	Concentration/flow rate	Sex	Baseline or control	Defined behaviors	Results	Reference
W	Gf	~17%	M	✓	✓	↑ line crossing (locomotor activity), ↑ rearing, ↑ nose to lid, ↑ escape behaviors, and ↑ ultrasonic vocalizations (mean range 22 ± 19 kHz)	^6^
W	Gf	17%	M	✓	✓	↑ line crossing (locomotor activity; 50% of the rats), ↑ rearing, ↑ nose to lid, ↑ escape behaviors (60% of the rats)	^7^
W	Pf	10%	✗	✓	✓	↔ in freezing/immobility, ↔ rearing	^32^
W	Pf	>97%	M/F	✗	Vague	↓ wall touching, ↑ wall climbing (rearing), ↔ vocalizations^a^	^9^
W	Pf	100%	M	✗	✗	↓ normal behavior, ↑ behavioral agitation and excitation, ↓ immobility/freezing, ↑ signs of asphyxia	^3^
SD	Gf	10%	M	✓	✓	↔ line crossing (locomotor activity), ↔ rearing, ↔ escape behaviors	^29^
SD	GF	~18%	F	✓	✓	↑ line crossing (locomotor activity), ↑ rearing, ↔ escape behaviors, ↔ freezing/immobility	^27^
SD	Gf_a_	20%	M	✓	Telemetric recordings	↑ locomotor activity, ↔ freezing/immobility	^24^
SD	Gf_a_	20%	M	✓	✗	↑ freezing /immobility	^30^
SD	Gf	23%	M	✓	✓	↑ line crossing, ↑ rearing	^28^
SD	Gf	30%	F	✓	✓	↑ ultrasonic vocalizations (median range 51 kHz)	^2^
SD	Gf	High but undefined	M	✓	✓	↑ wall climbing (rearing), ↑ activity, ↑ shaking (undefined), ↔ ultrasonic vocalizations	^26^
SD	Gf	30%	M/F	✗	✓	↑ rearing, ↔ jumping, ↔ digging^b^	^33^
SD	Pf	10%	✗	✓	✓	↔ freezing/immobility and ↓ rearing	^32^
SD	Pf	~75%	M	✗	Vague	↔ signs of distress, ↔ vocalization, ↔ escape behaviors, ↔ tail lashing	^31^
LE	Gf	High but undefined	M	✓	✓	↔ wall climbing (rearing), ↓ activity, ↑ shaking (undefined), ↔ ultrasonic vocalizations	^26^
LE	Pf	10%	✗	✓	✓	↑ freezing/immobility and ↓ rearing	^32^
F	Gf	35%^c^	M	✗	✗	↑ interest and curiosity, ↔ vocalizations, ↔ signs of pain	^10^
WK	Pf	10%	✗	✓	✓	↑ freezing/immobility, ↔ rearing	^32^
P-rat	Gf		M/F	✗	✓		^35^
		10%				↔ rearing, ↔ rearing (5 in), ↔ rearing (7 in), ↔rearing (5 in/rear)^d^	
		30%				↑ rearing, ↔ rearing (5 in), ↔ rearing (7 in), ↔rearing (5 in/rear)^d^	
		70%				↔ rearing, ↔ rearing (5 in), ↔ rearing (7 in), ↑rearing (5 in/rear)^d^	

Strain: *W* Wistar, *SD* Sprague Dawley, *LE* Long Evans, *WK* Wistar Kyoto, *F* Fisher, *BN* Brown Norway, *P-rat* alcohol preferring rats; delivery method: *Pf* pre-fill, *Gl* gradual fill, *GF*_*a*_ 20% CO_2_ challenge; concentration or flow rate: static concentration (%) or flow rate (% CO_2_ chamber vol. min^−1^); sex: *M* male, *F* female, ✗ unspecified; baseline: ✓ present, ✗ absent; defined behaviors: ✓ if a clear ethogram, vague if an unclear ethogram, ✗ absent; results: ↑ increase, ↓ decrease, ↔ no change in behavior or absent.

^a^Within the same study, two different results were found depending upon age: for young rats no change in activity but increase in stationary episodes, while old rats decreased both activity and stationary episodes. These authors concluded arrived to the same conclusion for both young and old rats.

^b^Behavior when exposed to CO_2_ in an induction chamber in comparison to exposure in the rat home cage.

^c^Flow rate was given as 6 l/min^−1^ and cage size unspecified, calculations were made based on the brand and type of the cage (Makrolon type III = ~17.2).

^d^Comparison between flow rates.

#### Escapable exposure

Here, we describe the behavioral responses of rats when exposure to CO_2_ can be avoided. One approach to assess emotions during CO_2_ exposure is through choice and motivational tests. This approach is based upon the “hedonic principle;” i.e., that animals are motivated to avoid undesired end states (e.g., potential harms, pain, etc.) and approach desired ones^37^.

Choice tests involve giving animals two or more alternative conditions (e.g., different agents or the same agent at different concentrations), and measuring the amount of time spent in each alternative as an expression of preference^4^. Studies have shown that rats prefer lower than higher concentrations of CO_2_. Rat spent between 36 and 51 s in a chamber prefilled with room air, and 2.1 and 0.7 s in chambers with 25.5% and 50.8% CO_2_, respectively^5^.

The strength of aversion can be measured by giving rats the ability to avoid exposure, with an added cost to the avoidance^38^. For inhalant agents, strength of aversion has been investigated through aversion- and approach-avoidance tests. In the aversion-avoidance test, the cost of avoiding the agent in a preferred dark compartment is exposure to an aversive brightly lit compartment. Using the aversion-avoidance test with a flow rate of 24% chamber vol. min^−1^, all rats left the dark chamber filling with CO_2_, escaping to the previously avoided bright chamber^39^.

In the approach-avoidance test, the cost of escaping to an agent free cage is the loss of a sweet reward. Rats are highly motivated to eat sweet rewards even when fed their regular diet at libitum^40^. When tested with different static concentrations of CO_2_ in the approach-avoidance apparatus, rats tolerated concentrations ranging from <1 to 10% CO_2_, entering the test chamber, eating the sweet rewards, and staying in the gas chamber for ~300 s. However, at 10% CO_2_ rats stopped eating ~20 s earlier than when tested with 5% CO_2_, indicating that although 10% CO_2_ is aversive, rats will tolerate exposure to obtain the sweet rewards. But the latency to leave the gas chamber decreased to 46 s at 15% CO_2_, and to 5 s with 20% CO_2_ (ref. ^41^).

Other studies using flow rates between 14 and 19% CO_2_ chamber vol. min^−1^, have shown that aversion is variable. In the aversion-avoidance test, latency to avoid CO_2_ ranged from 7 to 48 s between rats^39^. In the approach-avoidance test, the concentration avoided varied among individuals ranging from 11 to 18.6% CO_2_ (ref. ^7^). Variation among individuals in rat aversion to CO_2_ has shown to be consistent across exposures, within aversion^27^- and approach-avoidance^27,42^. These results support the idea that rats vary in CO_2_ reactivity. However, all aversion studies report that rats avoid CO_2_ before becoming ataxic or recumbent, even when fasted for 24 h (ref. ^40^).

These results (summarized in Table 2) indicate that onset of negative emotional states occurs ~10% CO_2_, although some rats are willing to tolerate exposure up to 18% CO_2_. These concentrations are consistent with the onset and peak of active behaviors during forced exposure^6^, and freezing behaviors reported using the 20% CO_2_ challenge^30^.

**Table 2:** Escapable exposure studies specifying test used, delivery methods, concentration or flow rate used, strain and sex, and summary of results. In all choice and aversion tests all rats avoided CO_2_ before loss of consciousness.

Strain	Sex	Test	Delivery method	Concentration/flow rate	Variables measured	Results	Notes	Reference
W	F	Choice	Pf	100% air	Total dwelling time	>35 s		^4^
				25.5% CO_2_		1.9 ± 0.6 s		
				34.9% CO_2_		0.8 ± 0.1 s		
				50.8% CO_2_		0.7 ± 0.1 s		
W	F	Choice	Pf	100% air	Total dwelling time	>36 s		^5^
				25.5% CO_2_		2.1 ± 0.5 s		
				34.9% CO_2_		1.0 ± 0.1 s		
				50.8% CO_2_		0.7 ± 0.2 s		
SD	M	Aversion-avoidance	Gf	32% O_2_	Total dwelling time	~90 s^a^		^39^
SD	M	Aversion-avoidance	Gf	24% CO_2_	Total dwelling time	19 ± 5 s and 24 ± 3 s	Depending on the level of illumination of the light chamber	^39^
SD	F	Aversion-avoidance	Gf	19% CO_2_	Latency to avoid CO_2_	33 ± 6 s and 35 ± 4 s	Two consecutive exposures	^27^
					CO_2_% avoided	14 ± 2% and 15 ± 1%		
W	M	Approach-avoidance	Pf	100% air	Total dwelling time	~ 240 s^a^		^41^
				5% CO_2_		~ 230 s^a^		
				10% CO_2_		~ 225 s^a^		
				15% CO_2_		46 s^b^		
				20% CO_2_		5 s^b^		
W	M	Approach-avoidance	Gf	17% CO_2_	CO_2_% avoided	18.4 ± 2%		^41^
W	M	Approach-avoidance	Gf	21% air	Latency to leave the chamber	270 ± 6 s		^8^
				3–27% CO_2_	Latency to avoid CO_2_	↓ from ~ 95 s ^a^ to ~ 28 s^a^	Depending on flow rate	
				3–27% CO_2_	CO_2_% avoided	14–16%^a^		
W	M	Approach-avoidance	Gf	17% air	Latency to leave the chamber	288 ± 2 s		^7^
				17% CO_2_	Latency to avoid CO_2_	40 ± 2 s	Average from three consecutive exposures	
W	M	Approach-avoidance	Gf	15% CO_2_ (Exp. 1)	CO_2_% avoided	16.3 ± 0.3%	Average from different levels of food deprivation	^40^
				15% CO_2_ (Exp. 2)		13.6 ± 0.3%	Average from different levels of food deprivation	
SD	F	Approach-avoidance	Gf	18% O_2_	Latency to leave the chamber	237 ± 27 s		^27^
				18.5% CO_2_	Latency to avoid CO_2_	23 ± 4 s and 28 ± 4 s	Two consecutive exposures	
					CO_2_% avoided	9 ± 2 % and 11 ± 1 %		
SD	F	Approach-avoidance	Gf	20% air	Latency to leave the chamber	420 ± 27 s	Average from three exposures	^42^
				Midazolam + 20% air		391 ± 28 s	Average from three exposures	
				20% CO_2_	Latency to avoid CO_2_	25 ± 2 s	Average from three exposures	
					CO_2_ % avoided	10.7 ± 1.14%		
SD	F	Approach-avoidance	Gf					^42^
				Midazolam + 20% CO_2_	Latency to avoid CO_2_	40 ± 3 s	Average from three exposures	
					CO_2_ % avoided	15.5 ± 1.41%		

Strain: *W* Wistar, *SD* Sprague Dawley; delivery method: *Pf* pre-fill, *Gl* gradual fill; concentration or flow rate: static concentration (%) or flow rate (% gas chamber vol. min^−1^); sex: *M* male, *F* female; *s* seconds; *results* mean ± standard error (unless specified otherwise); *E*xp. experiment.

^a^Average values estimated from a figure.

^b^Median values.

Human feelings of immobility (freezing), feeling paralyzed, desire to flee, and wanting to leave the room following inhalation of similar concentrations of CO_2_ (20–35% CO_2_)^43,44^, seem consistent with the rat defense behaviors reported above. Humans report feelings of anxiety at lower CO_2_ concentrations, and fear and panic at higher concentrations. For example, prolonged inhalation (20 min) of low CO_2_ concentrations (7.5%) elicits anxiety and induces hypervigilance in healthy volunteers^45^. This emotional experience is likely related to hypercapnia (rather than to hypoxia), since CO_2_ was administered to provide a normoxic gas mixture (containing ~21% O_2_)^46^. Increasing doses (e.g., double inhalation of 35% CO_2_), often induce panic attacks in healthy people^47^. These results provide some basis for suggesting that rat behavioral responses to CO_2_ may also be associated with the onset of negative emotional states related to feelings of anxiety, fear, or panic.

As reviewed above, the behavioral responses of rats vary between individuals. This variation has also been reported for human subjects. Human sensitivity to CO_2_ falls on a continuum panic disorder (PD) patients being the most sensitive^48^. In healthy humans, increases in fear and anxiety were reported in only 50% of the participants after a double inhalation of CO_2_ concentrations between 9 and 35% (ref. ^49^). When healthy humans inhale 20% CO_2_ for 20 s, 13% and 20% of the individuals experience modest or greater feelings of immobility and desire to flee, respectively^44^. With a double inhalation of 35% CO_2_, 47–68% healthy humans experience panic attacks^47^. In PD patients, a single inhalation of 35% CO_2_ induces panic in 43–94% of the individuals, depending on the subtype of PD^48^. These parallels in the human and rat literature are consistent with the idea that individual variation in rat behavioral responses to CO_2_ are associated with differences in the emotions elicited by this agent.

#### Exposure in other situations

CO_2_ is often used as an unconditioned stimulus in studies designed to induce negative emotional states in rodents. These studies support the conclusion that CO_2_ exposure is anxiogenic. In the Vogel test, two opposing motivations—gaining a reward versus avoiding a punishment—are used to assess the anxiolytic and anxiogenic effects of drugs. Food or water-deprived rats can choose to receive a reward (water or food) at the cost of receiving punishments (shocks); anxiogenic drugs suppress reward consumption and anxiolytics increase reward consumption^50^. Using the Vogel test, rats previously exposed for 60 s to CO_2_:O_2_ (35:65%) showed a similar response to that of rats treated with the anxiogenic benzodiazepine receptor ligand FG 7142; rats exposed to CO_2_ suppressed water licking by 40% relative to control rats^51^, indicating that CO_2_ exposure has an anxiogenic effect.

In the open field test, the tendency to avoid the central area and display thigmotaxis (locomotion close to the walls of the apparatus) is enhanced by anxiogenic drugs, while anxiolytics increase locomotion in the central areas of the arena^52^. In the social interaction test, anxiogenic drugs decrease the frequency of social interactions (e.g., sniffing, following, and grooming) while anxiolytics have the opposite effect^53^. After exposure to the 20% CO_2_ challenge, rats showed a 15% increase in thigmotaxis^34^ and a 50% decline in social interactions compared to rats exposed to air^24^.

Conditioning tests are also used to assess the aversiveness of a stimulus, and different variants of this test can be found. Potentially aversive stimuli can be paired with a neutral stimulus (Pavlovian conditioning), such as a neutral environment (place conditioning). Rats are then later exposed only to the paired stimulus, in absence of the aversive stimulus. Avoidance of the stimulus is an indicator of aversion, but if avoidance is restricted then immobility can be used as a measure of aversion^54^. Rats exposed to vanilla scent before 30 s of forced inhalation of different concentrations of CO_2_ (<1, 5, 35, or 100%), showed a conditioned response to vanilla 24 h later. Rats that inhaled <1% CO_2_ froze less than rats that inhaled higher concentrations. Rats exposed to 100% CO_2_ froze more and this conditioning resisted extinction relative to rats exposed to 5% CO_2_. These results indicate that CO_2_ can be used to condition anxiety in rats, and that the degree of behavioral response (freezing) and extinction reflect the severity of the experience^55^.

In summary, rat emotional states induced by CO_2_ inhalation are sustained after exposure, and CO_2_ acts as an unconditioned negative stimulus especially at higher concentrations (Table 3).

**Table 3:** Studies assessing the effects of CO_2_ post exposure, specifying test used, delivery methods, concentration or flow rate used, strain and sex, and a summary of results.

Strain	Sex	Test	Delivery method	Concentration/flow rate	Treatment	Results	Reference
SD	M	Vogel	Pf	35:65% (CO_2_:O_2_)	CO_2_	↓ liking (40%^a^)	^51^
					Alprazolam (0.5 mg/kg i.p.) + CO_2_	↑ liking^a^	
SD	M	Pavlovian conditioning	Pf	From 1 to 100% CO_2_	CO_2_ + vanilla odor	↑ of freezing episodes with CO_2_ concentration	^55^
SD	M	Open field	Gf_a_	20%	CO_2_	↑ 15% thigmotaxis^a^; ↑ fecal boli production^a^	^64^
SD	M	Open field	Gf_a_	20%	CO_2_	↑ 15% thigmotaxis^a^; ↑ fecal boli production^a^	^34^
SD	M	Social interaction	Gf_a_	20%		↓ 50% social interactions^a^	^24^
SD	M	Open field	Gf_a_	20%	CO_2_	↔ thigmotaxis and line crossing^a^	^75^
					Lorazepam + CO_2_		
					1 mg/kg i.p.	↓ line crossing^b^	
					0.5 mg/kg i.p.	↔ line crossing^b^	
					0.3 mg/kg i.p.	↔ line crossing^b^	
					0.1 mg/kg i.p.	↔ line crossing^b^	
SD	M	Social interaction	Gf_a_	20%	CO_2_	↓ social interactions^a^	^75^
					Lorazepam + CO_2_		
					1 mg/kg i.p.	↓ social interactions^b^	
					0.5 mg/kg i.p.	↔ social interactions^b^	
					0.3 mg/kg i.p.	↑ social interactions^b^	
					0.1 mg/kg i.p.	↑ social interactions^b^	

Strain: *SD* Sprague Dawley; delivery method: *Pf* pre-fill, *GF*_a_ 20% CO_2_ challenge; concentration or flow rate: static concentration (%) or flow rate (% CO_2_ chamber vol. min^−1^); sex: *M* male, *F* female; results: ↑ increase, ↓ decrease, ↔ no change in behavior or absent; *i.p.* intraperitoneal.

^a^Compared to control rats exposed to air.

^b^Compared to rats treated with vehicle.

### Physiological responses

Sympathetic responses in rats exposed to CO_2_ include increased blood pressure^31^ and bradycardia before the loss of righting reflex (LORR)^1^. In rats, arterial blood pressure increases during the 20% CO_2_ challenge^24^. Bradycardia has been reported for rats exposed to flow rates between 10 and 20% CO_2_ chamber vol. min^−1^ (refs. ^1,29^), and to the 20% CO_2_ challenge^24^. The cardiovascular response also correlates with changes in PaCO_2_ and pH (ref. ^34^). Altholtz et al.^56^ found that rats anesthetized with 70% CO_2_ showed increased plasma corticosterone levels after 30 min (but see ref. ^35^); similar results were found for rats exposed to 35% CO_2_ (ref. ^57^). In humans, inhalation of CO_2_ concentrations between 7 and 14% for 10–20 min induces an increase in minute ventilation, blood pressure, heart rate, plasma noradrenaline, and cortisol^58^. A single inhalation of 35% CO_2_ activates the HPA axis, and cortisol increases for ~30 min after exposure. In addition, blood pressure increases and heart rate decreases with exposure^43,59^.

The sympathetic and neuroendocrine responses to CO_2_ exposure indicate arousal likely reflective of negative valence. When taken together with the behavioral responses described in the previous section, we suggest that these physiological responses to CO_2_ exposure are associated with negative emotional states.

### Neurobiological responses

In this section, we review the effects of CO_2_ on activation of brain regions, and nuclei within regions, involved in fear and anxiety. Chemoreceptive areas of the brain implicated in the ventilatory response to hypercapnia (e.g., medullary raphe, retrotrapezoid nucleus, caudal medulla, nucleus tractus solitaries, etc.) have been previously reviewed^60,61^.

The amygdala is implicated in emotional responses to sensory inputs, and in generating the behavioral and physiological adaptive responses^62^. In rats exposed to 10% CO_2_, the medial and central amygdala show increased c-Fos expression. This increase is associated with augmented minute ventilation, breathing frequency, and tidal volume^63^. Johnson et al.^64^ found that rats exposed to the 20% CO_2_ challenge tended to increase c-Fos expression in the central amygdala, and this increase was related to increased fecal boli production (indicative of fear and anxiety) and thigmotaxis in the open field tests, but no effect of hypercapnia on c-Fos expression was detected in the medial and basolateral amygdala. Increased c-Fos expression in the central and basolateral amygdala has been found in high CO_2_-sensitive mice (i.e., mice that show a higher freezing responses to 5% CO_2_ than low responders) due to CO_2_ inhalation compared to that of mice exposed to air^65^. Asic1a^+/+^ mice (mice with intact acid sensing ion channels 1a in the amygdala and in other brain regions) froze when exposed to 10% static CO_2_, increased thigmotaxis when exposed to 20% CO_2_ in an open field test and preferred an air chamber, to a chamber prefilled with 20% CO_2_ in a choice test. These responses were attenuated in Asic1a^−^^/−^ mice (mice without intact acid sensing ion channels). In vitro amygdala neurons of Asic1a^+/+^, but not Asic1a^−/−^ mice, responded to a reduction in pH. In addition, CO_2_ inhalation decreased pH in the basolateral amygdala of Asic1a^+/+^ mice. Focal acidification of this region elicited a strong freezing response, while the administration of HCO_3_^−^ (to counteract acidosis) reduced freezing due to CO_2_ inhalation^66^. Overall, these results suggest that the amygdala acts as a chemoreceptor for changes in PaCO_2_/H^+^, and its activation is involved in the behavioral response to CO_2_.

The bed nucleus of the stria terminalis (BNST), frequently referred as the extended amygdala, is associated with modulation of behavioral responses to threatening stimuli^67^. Taugher and colleagues^68^ have shown that the BNST is also involved in the behavioral response to hypercapnia. These authors found that lesions in the BNST decreased freezing responses of mice during 10% CO_2_ exposure. When compared air exposure, inhalation of 5% CO_2_ increased c-Fos expression in the BNST of high CO_2_-sensitive mice^65^. In rats, c-Fos expression in the BNST did not differ when exposed to the 20% CO_2_ challenge versus air^64^.

Activation of the hypothalamus is related to the execution of different behavioral and physiological responses; the most commonly identified is the activation of the HPA axis and modulation of autonomic responses. During the HPA axis cascade, the paraventricular nucleus (PVN) in the hypothalamus secretes corticotropin-releasing factor^69^. Several studies indicate that when rats are exposed to CO_2_ concentrations of between 5 and 20%, the PVN shows a high density of positive c-Fos expression, indicating PVN activation during hypercapnia^64,70–72^. One other study reports no significant increase in c-Fos expression in the PVN of rats exposed to 10% CO_2_ (ref. ^63^).

The dorsomedial region of hypothalamus (DMH) is involved in the execution of behavior responses to aversive stimuli^73,74^. One study reported that rats exposed to 10% CO_2_ did not differ from controls in the number of labeled cells on the DMH^63^. However, other studies have found that the DMH shows high c-Fos expression in rats exposed to 5% CO_2_ and the 20% CO_2_ challenge^30,64,70^, particularly in orexin neurons^34^ found only in the DMH and perifornical nucleus of the hypothalamus^75^. Thigmotaxis in the open field was attenuated in rats treated with an orexin receptor antagonist before the 20% CO_2_ challenge^34^. Orexin neurons are chemosensitive; the firing rate of in vitro orexin neurons increases with fluctuations in CO_2_ and pH (ref. ^76^). Furthermore, individual differences in rat behavioral responses to the CO_2_ challenge were related to variation in orexin activity in the lateral hypothalamus^36^. These results show that the DMH and the perifornical nucleus of the hypothalamus are involved in the behavioral response to hypercapnia, and act as chemoreceptors.

Another region relevant in the modulation of behavioral responses to threatening stimuli is the periaqueductal gray (PAG). The ventrolateral PAG (VLPAG) is involved in immobility responses^77^, while the dorsal PAG (dorsolateral DLPAG and dorsomedial DMPAG) is related to flight behaviors^78,79^. The VL, DL, and DMPAG of rats exposed to CO_2_ show a dose-dependent increased c-Fos expression^75,80^. Rats exposed to CO_2_ concentrations between 8 and 13% show increased immobility and flight behaviors associated with PAG electrical stimulation^81^. Lesions in the DL and DMPAG of rats exposed to low concentrations of CO_2_ (7% CO_2_) decreased the ventilatory response compared to controls, without altering the cardiovascular response^82^. These results indicate that the PAG is activated during CO_2_ inhalation and involved in the behavioral and ventilatory responses to hypercapnia.

Consistent with this literature on rats (summarized in Table 4), research on human subjects shows that CO_2_ inhalation is associated with the activation of the amygdala, PAG, hypothalamus, and anterior insula. Moreover, this work on humans shows that activation of these brain regions is correlated with feelings of dyspnea^83^. Interestingly, patients with bilateral amygdala lesions still experienced fear and panic when inhaling 35% CO_2_ (ref. ^84^), but not when exposed to external life-threatening stimuli^85^.

**Table 4:** Neurobiological responses to CO_2_ specifying brain region, delivery method, concentration or flow rate used, and a summary of results. In all studies only male Sprague Dawley rats were used. All rats were forced exposed to CO_2_.

Brain region	Delivery method	Concentration/flow rate	c-Fos expression	Reference
Amygdala	Pf	10%		^63^
Medial			↑^a^	
Central			↑^a^	
Basolateral			↔	
Amygdala	Gf_a_	20%		^64^
Medial			~↑	
Central			↔	
Basolateral			↔	
BNST	Gf_a_	20%	↔	^64^
Hypothalamus	Pf	5%		^70^
PVN			↑	
DMH			↑	
Hypothalamus	^a^	15%		^71^
PVN			↑	
Hypothalamus	Pf	10%		^63^
PVN			↔	
DMH			↔	
Hypothalamus	Gf_a_	20%		^64^
PVN			↑	
DMH			↑	
Hypothalamus	Pf	5 and 12%	↑	^72^
PVN				
Hypothalamus	Gf_a_	20%		^34^
DMH			↑	
Periaqueductal gray	Gf_a_	20%		^36^
VLPAG			↑	
DLPAG			↑	
DMPAG			↑	
Periaqueductal gray	Pf	8, 10, and 15%		^81^^b^
VLPAG			↑	

Delivery method: *Pf* pre-fill, *GF*_*a*_ 20% CO_2_ challenge; concentration or flow rate: static concentration (%) or flow rate (% CO_2_ chamber vol. min^-1^); results: ↑ increase, ↓ decrease, ↔ no change, ~↑ tendency.

^a^Rats were ventilated.

^b^In this study, the stain and sex of the rats used are unspecified.

A key central chemoreceptor region is the medullary raphe; local acidification of the medullary raphe produces an increase in the ventilatory response^86^. Serotonin (5HT) is originated in the medullary raphe by tryptophan hydroxylation. Serotonin is implicated in mediating emotional states, perception, cognition, and sympathetic arousal^87^. Administration of serotonin reuptake inhibitors (SRIs), which increase synaptic serotonin, decreases anxiety in humans, and reduces respiratory rate of rats exposed to 6% CO_2_ (ref. ^88^). Mice treated with selective serotonin reuptake inhibitors (SSRIs) showed a decrease in freezing responses and ventilatory response during 5% CO_2_ exposure, and showed less context conditioning (rearing behavior) after exposure, than did control mice^89^. These results indicate that serotonin is involved in the ventilatory and behavioral response to hypercapnia. In humans, treatment with serotonin antagonists, or tryptophan depletion before one or two inhalations of 35% CO_2_, enhances the experience of anxiety, fear, dyspnea, and panic^90,91^. Treatment with serotonin precursors and SSRIs have the opposite effect^92,93^.

The role of the γ-aminobutyric acid (GABA)—a mammalian neurotransmitter that mediates synaptic inhibitions and is associated with anxiety^94^—is also involved in the response to hypercapnia. In rats, stressful events like acute handling^95^, chronic restraint^96^, and social isolation^97^ decrease GABA_A_ receptor function. The administration of compounds that bind to benzodiazepine recognition sites and that decrease the function of GABA_A_ receptors (i.e., anxiogenic drugs) induce anxiety-like behavior in rats^98^. Likewise, exposure to 35% CO_2_ decreases GABA_A_ function in the rat cerebral cortex, cerebellum, and hippocampus. The administration of benzodiazepines—which bind to the benzodiazepine receptor sites enhancing GABAergic transmission—before exposure to CO_2_, counteracts the effects of CO_2_ on GABA_A_ receptor functioning^99,100^, increase reward consumption in a Vogel conflict test^51^, increase tolerance to CO_2_ in approach-avoidance^42^, and enhance social interactions in the social test (at doses that do not impair locomotor activity)^75^. These results indicate that CO_2_ acts as negative stimulus on GABA_A_ functioning, and that following drug treatment with known anxiolytics the behavioral effect of CO_2_ is diminished. Similarly, healthy people pretreated with alprazolam before 5, 7.5, and 35% CO_2_ inhalation, show diminished experiences of fear, feeling like leaving the room, dyspnea, panic, and distress^101,102^.

In summary, brain areas, ion channels, and neurotransmitters involved in fear and anxiety are activated by hypercapnia. These regions include the amygdala, BNST, hypothalamus, and PAG. This body of evidence is consistent with the conclusion that rats experience negative emotions when inhaling CO_2_. Since these responses can, to some extent, be counteracted by the administration of anxiolytics and SRIs, and similarly to behavioral changes in rats, benzodiazepines, serotonin precursors, and SRIs ameliorate feelings of anxiety, fear, distress, dyspnea, and panic due to hypercapnia in humans.

## Conclusions

Concentrations <10% CO_2_ are tolerated, do not elicit intense behavioral responses, and cause mild conditioning in rats. Rats respond with active and passive defense behaviors to concentrations >10%. If escape is possible all rats avoid CO_2_, even to concentrations <10%, but when motivated some rats may tolerate higher concentrations (up to 18% CO_2_). The behavioral responses in the open field and social tests show that negative emotions, which resemble those associated with exposure to anxiogenic drugs, were sustained when CO_2_ was no longer present. Exposure to concentrations over 35% has anxiogenic effects and produces strong conditioning. The level of conditioning and extinction resistance depend upon CO_2_ concentration, implying that the magnitude of the emotional response increased with the intensity of the stimuli. The behavioral responses to CO_2_ are accompanied with neuroendocrine and sympathetic activation. Brain responses related to fear and anxiety are detected when rats inhale CO_2_. Overall, these studies indicate that CO_2_ elicit negative emotional states in rats.

Humans report feelings of fear, anxiety, dyspnea, distress, and panic during CO_2_ inhalation, and the evidence reviewed above suggests that rats experience similar emotions. Caution is required when functional homology is used to draw inferences regarding felt experiences^19^, but ventilatory and cardiovascular changes due to CO_2_ inhalation are similar between rats and humans, behavioral responses of rats when exposed to CO_2_ correspond well with feelings reported by humans following CO_2_ inhalation, and human feelings of anxiety, fear, dyspnea, and panic in response to hypercapnia can be attenuated by benzodiazepines and SSRIs (drugs that also diminish the defense behaviors seen in rats exposed to CO_2_). In addition, variation in rat and human CO_2_ reactivity is linked to the emotional experience during inhalation.

Throughout this review, we used functional homology to link rat and human felt emotions during CO_2_ inhalation. Although such inferences require caution, there is considerable research in human felt emotions due to CO_2_ inhalation. One example of a study comparing rodents and humans using the same CO_2_ concentrations is that by Leibold and colleagues^103^. These authors used static 9% CO_2_ concentrations to compare behavioral, respiratory, and cardiovascular responses of mice and humans. This study found that mice avoided the central areas of an open field, showed immobility responses and produced fecal boli during 9% CO_2_ exposure, and humans reported an increase in feelings of fear and panic due to 9% CO_2_ inhalation. Inhalation of 9% CO_2_ induced bradycardia in both species. The authors concluded that mice reactivity to CO_2_ can be used as model for humans. We suggest also that the opposite is also true; i.e., that human reports can be used to better understand the emotional experience of rodents during CO_2_ exposure.

Taken together, this evidence supports our conclusion that rats experience negative emotions during and after CO_2_ exposure, and that these emotions likely correspond to fear, anxiety, dyspnea, distress, and panic. This work contributes to the study of translational models of anxiety and panic, and is also relevant to ongoing debate regarding the use CO_2_ for killing animals. In addition, we suggest that this literature will be of interest to all who study felt emotions in animals.
